# Evaluation of Complications, Peripheral Neuropathic Pain, and Sleep Quality in Patients With Diabetes Mellitus

**DOI:** 10.1002/brb3.70605

**Published:** 2025-05-30

**Authors:** Dilek Yildirim, Deniz Aras

**Affiliations:** ^1^ Faculty of Health Sciences, Department of Nursing İstanbul Aydin University Istanbul Turkey; ^2^ Diabetes Center Başakşehir Çam and Sakura City Hospital Istanbul Turkey

## Abstract

**Background:**

Most patients with diabetic peripheral neuropathy (DPN) suffer from problems such as neuropathic pain, sleep disturbance, anxiety, and depression, which negatively affect their quality of life. Painful DPN is one of the most common grounds for seeking medical attention. The aim of this study is to evaluate the complications, peripheral neuropathic pain, and sleep quality in diabetic patients.

**Methods:**

This descriptive, cross‐sectional, and correlational study included 300 patients with neuropathic pain. Data were obtained using the Descriptive Information Form, Self‐Administered Leeds Assessment of Neuropathic Symptoms and Signs Scale (S‐LANSS), and Richards–Campbell Sleep Questionnaire (RSQ).

**Results:**

The mean level of peripheral neuropathic pain that the patients suffered in the last week, according to the VAS, was 4.143 ± 2.983. The S‐LANSS mean score of the patients was 16.493 ± 7.536, and their total mean score on the RSQ was 39.986 ± 33.150. There was a statistically significant negative correlation between the mean scores of the S‐LANSS and the RSQ (*r* = −0.489, *p* < 0.001). There was also a statistically significant negative correlation between the mean scores of the Neuropathic Pain VAS and the RSQ (*r* = −0.401, *p* < 0.001). Total S‐LANSS Score, duration of diabetes diagnosis, HbA1c%, neuropathic pain VAS severity, and age accounted for 36.1% of the variance in the quality of sleep score of patients.

**Conclusion:**

Neuropathic pain and poor sleep quality were prevalent in diabetic patients. It was found that the duration of DM and microvascular complications, particularly neuropathy, impaired sleep quality.

## Graphical abstract

Neuropathic pain and poor sleep quality were prevalent in diabetic patients. It was found that the duration of DM and microvascular complications, particularly neuropathy, impaired sleep quality. Age, diabetes duration, and neuropathic pain severity were strongly correlated with poorer sleep quality.

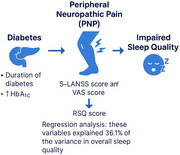

## 1 Introduction

Diabetes mellitus poses a major health challenge, particularly in developing countries, where its prevalence is rising and leading to serious complications such as neuropathy, retinopathy, and nephropathy (Mekuria Negussie and Tilahun Bekele 2024). Diabetic peripheral neuropathy (DPN) is frequently seen in diabetic patients and accounts for a significant portion of the costs related to diabetes care. Its impact on quality of life and the financial burden it creates highlight the importance of effective management and prevention strategies for this complication (Wang et al. 2023). It is estimated that DPN impacts approximately 50% of all individuals with diabetes worldwide. DPN leads to considerable morbidity, reduces the quality of life, and is linked to a higher risk of mortality (Iqbal et al. 2018).

Diabetic neuropathy encompasses a range of clinically varied disorders that impact the nervous system, characterized by different anatomical features, clinical presentations, and phenotypes. A common underlying pathophysiological mechanism involves hyperglycemia and microangiopathy (Iqbal et al. 2018; Wang et al. 2023). The most prevalent form is distal symmetric sensorimotor polyneuropathy; however, complications can arise across various body systems due to the involvement of autonomic nerves (Alobaid et al. 2025; Iqbal et al. 2018). Multiple studies have indicated that various risk factors associated with the development of DPN include age, level of education, duration of diabetes, smoking, inadequate glycemic control, hypertension, and hyperlipidemia (Choi et al. 2021; Fu et al. 2024; Won et al. 2017). Although DPN imposes a considerable economic burden on healthcare systems and affects quality of life, treatment options are scarce, emphasizing the importance of prevention as the primary objective (Iqbal et al. 2018).

Many individuals with DPN experience issues such as neuropathic pain, sleep disturbances, anxiety, and depression, all of which adversely impact their quality of life. The pain is often described as electric shock‐like, sharp, tingling, burning, or throbbing. It usually begins in the feet and can spread to the calves, fingers, and hands, following a “sock and glove” distribution. Chronic pain is a personal experience that affects cognitive and emotional factors, disrupts mood and thought processes, causes functional limitations, and often interferes with everyday activities. Additionally, sleep issues, chronic pain, and mood disorders frequently coexist and are believed to influence each other in interconnected ways (Ekici et al. 2017; Gore et al. 2005; Mekuria Negussie and Tilahun Bekele 2024).

Previous research has shown that the painful sensations associated with DPN can result in sleep disturbances, and more intense pain is linked to a decline in sleep quality (Choi et al. 2021; Fu et al. 2024; Naranjo et al. 2019; Won et al. 2017). Painful DPN is one of the primary reasons individuals seek medical care. This form of neuropathic pain frequently leads to challenges with falling asleep, pain‐related sleep disruptions, burning sensations, and itching. The resulting sleep loss can contribute to anxiety and depression, further worsening sleep disturbances, causing many patients to enter a detrimental cycle of sleep deprivation. Consequently, this lack of sleep leads to decreased energy levels, significantly impacting the patient's ability to function and reducing their independence in daily activities (Fu et al. 2024; Wu et al. 2021).

Diabetes mellitus is a chronic disease associated with multiple complications, such as DPN and impaired sleep quality. These complications can significantly impact patients’ daily functioning and overall quality of life. While several studies have investigated these factors individually, only a limited number of studies have assessed them concurrently within the same patient population. In particular, the interaction between PNP and sleep disturbances, and their combined influence on patients’ quality of life and disease management, remains underexplored. Furthermore, identifying the variables that affect sleep quality through multiple regression analysis is crucial for better understanding these complex relationships and guiding targeted interventions. This study aims to evaluate the complications, peripheral neuropathic pain, and sleep quality in diabetic patients.

## 2 Material and Methods

### 2.1 Setting and Design

This descriptive, cross‐sectional, and correlational study was carried out in the diabetes outpatient clinic of a city hospital in Istanbul, Turkey from July 2023 to February 2024. The study was conducted in accordance with the STROBE guidelines, which aim to standardize the reporting of observational research (von Elm et al. 2007).

### 2.2 The Study Population and Sample

The study comprised patients who chose to participate voluntarily and were 18 years of age or older, diagnosed with diabetes mellitus for at least 1 month, and diagnosed with neuropathic pain by a physician. Patients with communication problems (visual and hearing impairment) and patients with a psychiatric illness were excluded from the study. The G‐Power 3.1.9.7 program was utilized to determine the minimum sample size necessary for the study. Based on calculations, a minimum sample size of 300 was required, considering a medium effect size (*d* = 0.5), a margin of error of 5% (*α* = 0.05), and a power of 80% (*β* = 0.80) (Faul et al. 2007).

### 2.3 Data Collection

After their examination in the outpatient clinic, the patients completed the questionnaires. The researcher collected all the questionnaires using a questionnaire form and the face‐to‐face interview method. In addition, other complications (retinopathy, nephropathy, peripheral vascular disease, amputation, etc.) and information about the disease were obtained from hospital records and physician reports. To avoid bias, the researcher who collected the data did not participate in the data analysis process. After providing instructions on how to fill out the questionnaires, we left the patients alone to do so. The researcher read the questions to illiterate people and recorded the patient's responses. This way is preferred to make them feel comfortable and to answer easily.

### 2.4 Data Collection Tools

Data were gathered through the Descriptive Information Form, the Self‐Administered Leeds Assessment of Neuropathic Symptoms and Signs Scale (S‐LANSS), and the Richards–Campbell Sleep Questionnaire (RSQ).

### 2.5 Descriptive Information Form

This form includes the sociodemographic characteristics of the patients and general information about their diseases. The form consists of a total of 14 questions that the researcher prepared based on the literature. The form includes questions about demographic characteristics (age, gender, marital status, educational level, etc.) and disease‐related characteristics (history of chronic disease, duration of diabetes, mode of diabetes treatment, etc.).

### 2.6 Self‐Administered Leeds Assessment of Neuropathic Symptoms and Signs Scale

This is a brief, multidimensional scale designed for bedside assessment based on questionnaire data. It is particularly effective in differentiating between neuropathic and nociceptive pain. The LANSS comprises seven items: five questions focus on pain symptoms, while the remaining two relate to sensory evaluation through allodynia and a pin‐prick test. Scores range from 0 to 24, with a score of 12 or higher indicating neuropathic pain. If the score is 12 and above, it is classified as neuropathic pain. The questions are formatted for yes‐or‐no responses. The S‐LANSS was developed to address the need for clinical examination in the original LANSS (Bennett 2001). Koc and Erdemoglu conducted a study to assess the Turkish validity and reliability of the S‐LANSS neuropathic pain scale. Their research aimed to ensure that the scale is suitable for use in Turkish‐speaking populations, providing a reliable tool for evaluating neuropathic pain in clinical settings (Koc and Erdemoglu 2010). This study revealed that Cronbach's alpha internal consistency was 0.820.

### 2.7 Richards–Campbell Sleep Questionnaire

In 2015, Özlü and Özer conducted a study to evaluate its reliability and validity in a Turkish context. Participants rate each item on a scale from 0 to 100. The scale was developed by Richards and consists of six items (Richards et al. 2000). Notably, Item 6, which assesses noise levels in the environment, is excluded from the overall score calculation. The final score is determined by averaging the responses to the other items. Scores ranging from 0 to 25 indicate *very poor sleep*, while from 76 to 100 reflect *very good sleep*. The scale's range extends from 0 to 100, where higher scores signify better sleep quality. The internal consistency of the scale was established with a Cronbach's alpha of 0.91, and this study found an improved alpha of 0.921, indicating strong reliability.

### 2.8 Data Analysis

Data collected for the study were analyzed using SPSS for Windows (Version 26.0). Descriptive statistics for continuous variables were reported as means and standard deviations, while categorical variables were shown as frequencies and percentages. There were no missing data, with a total of 300 participants included in the analysis. The Kolmogorov–Smirnov test was employed to assess the normality of the data distribution, indicating a non‐normal distribution. The Kruskal–Wallis *H* test was applied to compare four independent groups, and the Student's *t*‐test was used for comparisons between two independent groups. The correlation between participants’ mean scores on the S‐LANSS and the RSQ was examined using the Pearson correlation test. Multiple linear regression analyses were conducted to evaluate the predictors of sleep quality, focusing on the total S‐LANSS score, duration of diabetes diagnosis (years), HbA1c%, neuropathic pain VAS severity, and age. Variable selection for the regression model was guided by the results of the multicollinearity test, which assessed the total S‐LANSS score, duration of diabetes, HbA1c%, neuropathic pain severity, and age using the variance inflation factor (VIF), and tolerance. Variables with a tolerance value above 0.2 and a VIF below 10 were included in the analyses. No multicollinearity was found among the variables. Consequently, fear of hypoglycemia, adherence to treatment, duration of Type 2 diabetes mellitus (T2DM), and age were incorporated into the regression analysis. The threshold for significance was established at 0.05 (Tabachnick and Fidell 2014).

### 2.9 Ethics Statement

To conduct the study, written approval was obtained from the Istanbul Aydin University Ethics Committee (dated April 5, 2023, numbered 2023/04) along with institutional permission from the hospital where the research took place (dated June 20, 2023, numbered 2023/11). Prior to the study's implementation, participants were thoroughly informed about the research process and the data collection tools, and their consent was obtained. The study adhered to the principles outlined in the Declaration of Helsinki.

## 3 Results

The mean age of the patients was 56.440 ± 12.712 years, and more than half (*n* = 170, 56.7%) of them were female. Most of the patients (88.7%, *n* = 266) were married, and 39% (*n* = 117) were primary school graduates. The mean duration of diabetes for the patients was 13.923 ± 8.826 years. The mean HbA1c of the patients was 11.717 ± 5.802, and 70% (*n* = 210) of the patients also suffered from hypertension. 47.3% (*n* = 142) of the patients reported that they were on insulin therapy only, and 27.7% (*n* = 83) did not measure their blood glucose at home. Total 86.3% (*n* = 259) of the patients were amputated due to retinopathy, 15.3% (*n* = 46) due to nephropathy, 7.3% (*n* = 22) due to peripheral vascular disease, and 1% (*n* = 3) due to diabetes‐related complications (Table 1).

**TABLE 1:** Sociodemographic information and disease‐related characteristics of patients (*n* = 300).

Variables	Mean ± SD	Min.–Max.
Age (years)	56.440 ± 12.712	18–88
Gender	*n*	%
Female	170	56.7
Male	130	43.3
Marital status		
Married	266	88.7
Single	34	11.3
Educational status		
Illiterate	58	19.3
Literate	57	19.0
Primary education	117	39.0
High school	39	13.0
University	26	8.7
Postgraduate	3	1.0
	Mean ± SD	Min.–Max.
Diabetes diagnosis time (years)	13.923 ± 8.826	1–40
HbA1c, %	11.717 ± 5.802	6.0–12.1
Comorbidities		
Hypertension	210	70.0
COPD	4	1.3
Heart failure	10	3.3
Kidney failure	4	1.3
Others	18	6.0
None	54	18.0
Diabetes treatment		
Oral antidiabetic only	59	19.7
Insulin only	142	47.3
Oral antidiabetic and insulin together	99	33.0
Blood sugar measurement at home		
Yes	217	72.3
No	83	27.7
Complications due to diabetes		
Yes	300	100.0
Retinopathy		
No	41	13.7
Yes	259	86.3
Nephropathy		
No	254	84.7
Yes	46	15.3
Peripheral vascular disease		
No	278	92.7
Yes	22	7.3
Amputation		
No	297	99.0
Yes	3	1.0

Abbreviations: COPD, chronic obstructive pulmonary disease; SD, standard deviation.

The mean level of peripheral neuropathic pain experienced by patients over the past week, as measured by the Visual Analog Scale (VAS), was 4.143 ± 2.983. The average score on the S‐LANSS was 16.493 ± 7.536, while the total mean score on the RSQ was 39.986 ± 33.150 (Table 2).

**TABLE 2:** VAS Pain, S‐LANSS, and Richards–Campbell Sleep Scale mean scores of diabetic patients (*n* = 300).

Scales	Mean ± SD
VAS Pain level	4.143 ± 2.983
S‐LANSS	16.493 ± 7.536
Richards–Campbell Sleep Scale total score	39.986 ± 33.150

Abbreviation: SD, standard deviation.

A statistically significant negative correlation was found between the average scores of the S‐LANSS and the RSQ (*r* = −0.489, *p* < 0.001). Additionally, there was a significant negative correlation between the average scores of the Neuropathic Pain VAS and the RSQ (*r* = −0.401, *p* < 0.001; Table 3).

**TABLE 3:** The relationship between VAS Pain, S‐LANSS, and Richards–Campbell Sleep Scale mean scores of diabetic patients (*n* = 300).

Variables	1	2
1. Neuropathic pain VAS severity	1.000	
2. Total S‐LANSS score	0.595^**^	1.000
3. Richards–Campbell Sleep Scale total score	−0.401^**^	−0.489^**^

** *p* < 0.01.

The multiple regression analysis showed that the quality of sleep scores of the patients significantly predicted total S‐LANSS score, Duration of diabetes diagnosis (years), HbA1c%, neuropathic pain VAS severity, and age (*F* = 25.878, *p* < 0.001). Total S‐LANSS score, duration of diabetes diagnosis (years), HbA1c%, neuropathic pain VAS severity, and age accounted for 36.1% of the variance in the quality of sleep score of patients (Table 4).

**TABLE 4:** Multiple regression analysis of variables that affect the quality of sleep (*n* = 300).

	*B*	Standard error	Standard beta (β)	*t*	*p*	95% CI
Constant	436.103	36.926		11.810	< 0.001	
Total S‐LANSS score	−6.956	1.172	−0.406	−5.933	**< 0.001**	−9.266 to −4.646
Duration of diabetes diagnosis (years)	1.935	0.923	0.133	2.096	**0.037**	0.116 to 3.754
Age (years)	−1.819	0.625	−0.181	−2.911	**0.004**	−3.051 to −0.588
HbA1c, %	−1.262	1.170	−0.057	−1.078	0.282	−3.566 to 1.043
Neuropathic pain VAS severity	−8.549	2.989	−0.212	−2.860	**0.005**	−14.439 to −2.660
Dependent variable: Richards–Campbell Sleep Scale total score						
*R *= 0.601 *R* ^2^ = 0.361 Adjusted *R* ^2^ = 0.347 *F *= 25.878 *p* < 0.001 Durbin Watson = 1.937 (1.5–2.5), VIF = 0.1–10						

Values in bold indicate statistical significance at *p* < 0.05

## 4 Discussion

This study examined the correlation between complications, peripheral neuropathic pain, and sleep quality in diabetic patients.

The study found that the average HbA1c level among the patients was 11.717, and all participants experienced at least one complication related to diabetes. The most prevalent complications included neuropathy, retinopathy, and nephropathy, respectively. Consistent with the current study, existing literature indicates that diabetic nephropathy and retinopathy impact about 25% of individuals with Type 2 diabetes mellitus, while diabetic neuropathy is observed in 50% of those with diabetes (Faselis et al. 2020). A study conducted in China reported that the prevalence of DPN was 67.6%. The results of this study also include the prevalence of DPN increased with age and course of diabetes and decreased with body mass index (BMI) and education level (Wang et al. 2023).

One of the major findings of the present study was that as the patients’ level of diabetes‐related neuropathic pain rose, their sleep quality worsened. A study conducted with 156 patients with Type 2 diabetes mellitus in a hospital in South India examined the correlation between diabetic neuropathic pain that patients suffered from and their sleep quality and reported a high prevalence of sleep disturbance in patients with diabetic neuropathy (Lawrence et al. 2022). A study conducted in China with 193 patients examined sleep quality in Type 2 diabetic patients with DPN and showed that patients had poor sleep quality, and their quality of life also worsened (Fu et al. 2024). Another study reported a significant increase in the incidence of sleep apnea syndrome in patients with DPN compared to diabetic patients without diabetic neuropathy and healthy individuals (*p* < 0.0001) (Bahnasy et al. 2018). Meta‐analysis study that examined the correlation between obstructive sleep apnea and diabetic neuropathy reviewed 11 studies, including 1842 patients. The meta‐analysis resulted in a significant correlation between obstructive sleep apnea and diabetic neuropathy (Gu et al. 2018). Another systematic review showed that sleep apnea–hypopnea syndromes were correlated with diabetic neuropathy (Abelleira et al. 2021). Diabetic patients with chronic neuropathic pain suffer from lower quality of life, and higher levels of sleep disturbances, anxiety, and depression compared to patients without pain. Total 28 findings of the present study are compatible with the data in the literature.

The present study found that total S‐LANSS score, duration of diabetes diagnosis (years), HbA1c%, neuropathic pain VAS severity, and age accounted for 36.1% of the variance in patients’ sleep quality score. In a similar vein, a study on adults with Type 2 diabetes (*n* = 90; 52.2% female, mean age 57.4 years) revealed that variables of gender, HbA1c, neuropathic pain and fatigue, age, duration of diabetes, depressive symptoms, and distress accounted for 52% of the variance in sleep disturbance (Zhu et al. 2018). In a separate study, regression analysis indicated that neuropathy was an independent factor associated with poor sleep quality (OR 1.362, 95% CI 0.032–2.692, *p* = 0.045) (Öztürk et al. 2015).

In our study, a multiple regression analysis was conducted to identify the factors influencing sleep quality. The results indicated that HbA1c% (*β* = −0.057, *p* = 0.282) did not significantly affect sleep quality, suggesting that HbA1c% might not have a direct impact on sleep quality. This implies that the relationship between HbA1c% and sleep quality may need to be examined in conjunction with other factors, and further research is needed to better understand its role. These findings highlight the importance of considering multiple factors when evaluating sleep quality in diabetic patients, as several variables can interact to influence overall sleep health and sleep quality.

### 4.1 Limitations

The study was conducted in patients with diabetes for more than 13 years, which is the strength of this study. Also, the study was performed with a large sample size for long‐term diabetic patients. The study was conducted in a single center. It is a limitation in generalizing the results of the study. As a cross‐sectional study, it is not possible to infer a causal relationship between the factors and the outcome variable.

## 5 Conclusions

Results of the present study showed that neuropathic pain and poor sleep quality were prevalent in diabetic patients. It was found that the duration of DM and microvascular complications, particularly neuropathy, impaired sleep quality. Based on previous studies, the researchers contributed to the current knowledge on the correlation between diabetes‐related symptoms, neuropathic pain, and sleep. It was found that age, diabetes duration, and neuropathic pain severity were strongly correlated with poorer sleep quality. Based on the study's limitations, further studies are required to determine the causality between sleep, neuropathic pain, and other diabetes complications.

## Author Contributions

**Dilek Yildirim**: conceptualization, investigation, funding acquisition, writing–original draft, writing–review and editing, visualization, validation, methodology, software, formal analysis, project administration, resources, supervision, data curation. **Deniz Aras**: conceptualization, investigation, funding acquisition, writing–original draft, methodology, validation, visualization, writing–review and editing, software, formal analysis, data curation, supervision, resources.

## Conflicts of Interest

The authors declare no conflicts of interest.

### Peer Review

The peer review history for this article is available at https://publons.com/publon/10.1002/brb3.70605.

## Data Availability

The datasets that support the findings of this study are available from the corresponding author on reasonable request.
